# Development of an Organ-on-a-Chip-Device for Study of Placental Pathologies

**DOI:** 10.3390/ijms21228755

**Published:** 2020-11-19

**Authors:** Babak Mosavati, Andrew V. Oleinikov, E. Du

**Affiliations:** 1 Department of Ocean and Mechanical Engineering, College of Engineering and Computer Science, Florida Atlantic University, Boca Raton, FL 33431, USA; bmosavati2017@fau.edu; 2 Department of Biomedical Science, Charles E. Schmidt College of Medicine, Florida Atlantic University, Boca Raton, FL 33431, USA; aoleinikov@health.fau.edu; 3 Department of Biological Sciences, Florida Atlantic University, Boca Raton, FL 33431, USA

**Keywords:** placenta-on-a-chip, glucose transport, molecular concentration distribution, microfluidics

## Abstract

The human placenta plays a key role in reproduction and serves as a major interface for maternofetal exchange of nutrients. Study of human placenta pathology presents a great experimental challenge because it is not easily accessible. In this paper, a 3D placenta-on-a-chip model is developed by bioengineering techniques to simulate the placental interface between maternal and fetal blood in vitro. In this model, trophoblasts cells and human umbilical vein endothelial cells are cultured on the opposite sides of a porous polycarbonate membrane, which is sandwiched between two microfluidic channels. Glucose diffusion across this barrier is analyzed under shear flow conditions. Meanwhile, a numerical model of the 3D placenta-on-a-chip model is developed. Numerical results of concentration distributions and the convection–diffusion mass transport is compared to the results obtained from the experiments for validation. Finally, effects of flow rate and membrane porosity on glucose diffusion across the placental barrier are studied using the validated numerical model. The placental model developed here provides a potentially helpful tool to study a variety of other processes at the maternal–fetal interface, for example, effects of drugs or infections like malaria on transport of various substances across the placental barrier.

## 1. Introduction

Placenta is a critical organ that supports embryonic growth by mediating, among other things, nutrient and oxygen supply to the fetus as well as removing waste products and carbon dioxide from the fetus [1,2,3]. Insufficient placental development may affect those functions and, in turn, increase mortality and morbidity of both fetus and mother. It has been shown that insufficient oxygen and nutrients transport across the placental barrier causes fetus adverse outcome [4]. Malarial infection in placenta during pregnancy, known as placental malaria, causes severe clinical conditions such as low birth weight [5], premature birth [6], intrauterine growth restriction [7], or abortion [8]. Studying diffusion efficiency across the placental barrier will provide a better understanding of the pathophysiological mechanisms involved in poor outcomes associated with diseases such as malaria infections in pregnancy. However, study of human placental pathology remains a major experimental challenge as placenta is not easily accessible and highly species-specific on both host and pathogen sides, limiting the use of small animal models.

To study placenta, in vivo [9,10,11,12], ex vivo [13,14,15,16], and in vitro [17] models have been developed. In vivo investigations using animal models have encountered challenges in direct transferring the results from animals to humans due to difference in placenta anatomy [18]. This makes it challenging to correlate the experimental results on placentas of humans and other mammals. The ex vivo models have been used to study the placenta and the effects of the pharmaceutical drugs on the placental barrier. Ex vivo studies are conducted on a live organ outside of host. For example, ex vivo studies on human placenta have been conducted immediately after childbirth [19]. The ex vivo model is advantageous as it does not cause severe damage to the maternal vasculature [20] but is limited by the difficulties in technical placental perfusion set-up, challenges in acquiring permission from women to participate in studies, and ethical issues. In addition, it is challenging to handle chemical screening and testing on a wide scale [21].

Recently, organ-on-a-chips enabled by bioengineering and microfluidics-based approaches for mono and multicell cultures in 3D microfabricated devices have been developed to mimic various biological and pathological processes in human body, such as liver [22], bone [23], kidney [24], lung [25], heart [26], brain [27], and multiple organs [28]. These organ-on-a-chip models have provided important insights into physiological and pathological processes in human organs [29] as well as powerful platform for accelerated in vitro drug screening and testing [30]. Among the various human organs, placenta is one of the most complicated organs to replicate as it develops during pregnancy and its functionality and structure changes over the gestation period.

In order to address the challenges in studying human placenta, organ-on-a-chip technology has been utilized to recreate key functional units of this vital organ. Cultures of trophoblast and endothelial cells within the microfabricated devices have shown potentials to reconstruct and experiment on the transport mechanisms across the placental barrier. Yin et al. [31] developed a placenta microdevice platform where the maternal and fetal channels were designed in parallel on one layer of Polydimethylsiloxane (PDMS). This in vitro model was utilized to analyze placental responses, such as oxidative stress and barrier permeability to exposure of nanoparticles (NPs). Zhu et al. [32] used a microfluidic co-culture model to investigate the placental inflammatory responses to bacterial infection. Miura et al. [33] developed a microdevice replicating human placental barrier microvilli surface in vitro. The results demonstrated that the fluid shear activates microvilli formation in human placental trophoblastic cells. Blundull et al. [34] used co-culture model and investigated glyburide diffusion and heparin across the human placental barrier. The in vitro result of the glyburide transport rate was found to be similar to that measured in ex vivo human placenta techniques. Mandt et al. [35] studied trans-cellular transport process across this barrier and demonstrated differential permeability for the small molecules (350 Da riboflavin) with a size of glucose that can diffuse freely and large molecules (200 kDa dextran) that cannot pass this barrier. Pemathilaka et al. [36] studied the rate of caffeine transport across the placental barrier. A variety of techniques to model the placental barrier and their applications have been reported in this effort. See reference [37] for a comprehensive review.

Although the placenta-on-a-chip technology has been widely investigated to test transport of drugs and nutrients across the placental barrier, there are still some challenges, regarding the chip performance, maintenance of confluent trophoblast cells and screening the drugs in the human placenta in a rapid, cost-effective and systematic manner. In this paper, we demonstrated an experimental-numerical strategy to evaluate a microfluidics-based placenta-on-a-chip model in the study of mass transport across the placental barrier. First, we develop an experimental model and demonstrate its capability by determining glucose transport across the placental barrier. This device enables us to measure glucose diffusion across the barrier by co-culturing the human umbilical vein endothelial cells (HUVECs) and BeWo cells on a thin polycarbonate membrane to mimic a physiological placental barrier in vitro. Then, a 3D numerical model based on COMSOL Multiphysics software is developed and validated by comparing the simulation results to the diffusion rates measured in experiments. Further, we utilize this numerical model to investigate the glucose transport dynamics across the placental barrier under different conditions such as flow rate and membrane porosity.

## 2. Modeling and Simulation

### 2.1. Analytical Model and Governing Equations

**Figure 1:**
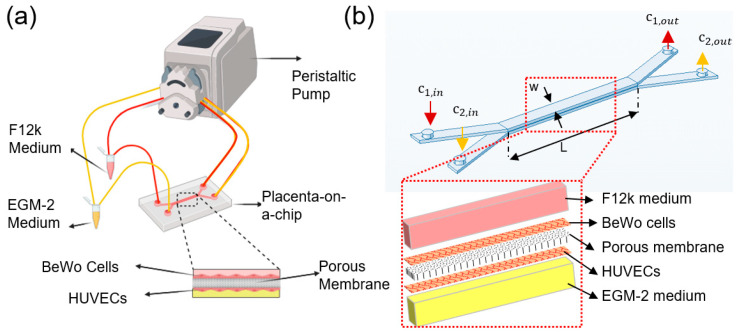
Glucose transfer analysis in the microfluidics-based modeling of human placental barrier: (**a**) schematic of the experimental setup, (**b**) geometry of the placenta-on-a-chip device with inset showing the exploding view of the multiple layers for finite element simulation.

The cross-sectional geometry of the placental bilayer microdevice is shown in Figure 1b. The width (w) and length (L) of micro channels are 1 mm and 1 cm, respectively. The channels height (H) and the membrane thickness are 200 µm and 10 µm, respectively. There is a balance between two types of mass transport in each channel, convection, and diffusion. The liquid flow convection is modeled based on the incompressible Navier–Stokes equation,

(1) $$\rho \left[\frac{\partial U}{\partial t}+U\cdot \nabla U\right]=-\nabla P+\mu \Delta U+\rho g$$

where ρ is the fluid density, *t* is time, *U* is the velocity vector field, *P* is the pressure, *μ* is the fluid viscosity, and g is gravity vector. Since, the flow velocity in both channels are small, inertial force can be neglected. Thus, fluid flow can be considered as the flow that passes through the porous membrane, following Darcy’s law,

(2) $$U=-\frac{K}{\mu }\nabla P$$

and

(3) $$K=\frac{{d^{2}}_{p}\varepsilon^{3}}{180{\left(1-\varepsilon \right)}^{2}}$$

where *K* is the material permeability, *µ* is dynamic viscosity, $d_{p}$ is particle diameter ($d_{p}$ = 1 nm for glucose molecules). Parameter ε is the membrane porosity, defined as the total volume of pores divided by the total volume of the membrane (ε=0.01 for the polycarbonate membrane, as provided by the manufacturer). The concentration distribution of given species is governed by convection-diffusion equation,

(4) $$\frac{\partial C_{G}}{\partial t}+\left(U\cdot \nabla C_{G}\right)=D\nabla^{2}C_{G}$$

where *D* is the diffusion coefficient of the species.

Cells consumes nutrients such as glucose to survive and proliferate. Michaelis-Menten kinetics was used to model cell consumption of glucose [38],

(5) $$\frac{\partial C_{G}}{\partial t}=D\nabla^{2}C_{G}-\left(U\cdot \nabla C_{G}\right)-\frac{V_{max.G\;}NC_{G}}{K_{mG}+C_{G}}$$

where $V_{max.G\;}$ is the maximum uptake rate, being 1.8 µmol/$10^{6}$ cells/d for HUVECs [39] and 1.1 µmol/$10^{6}$ cells/d for BeWo cells (40). $K_{mG}\;$ is the Michaelis-Mentenconstant, being 123 µmol/L and 1.5 mmol/L for HUVECs and BeWo cells, respectively [40,41]. The cell density and the concentration are shown by N (cells/cm^3^) and $C_{G}$, respectively.

The porous medium model was used to model the membrane separating the two channels. The porosity was modeled by scaling the molecular diffusivity by the void area created by the membrane pores. Stoke-Stein equation was used to estimate diffusion coefficients,

(6) $$D=\frac{K_{B}T}{6\pi \eta r}$$

where $r={\left(\frac{3M}{4\pi N}\right)}^{\frac{1}{3}}$, *K_B_* is the Boltzmann constant ($1.38\times 10^{-23}\;m^{2}kgs^{-2}k^{-1}$), *T* is the absolute temperature (*T* = 298 K), and r is the radius calculated based on the molecular weight, M, and the Avogadro number, N [32].

### 2.2. Numerical Simulation

3D simulation was carried out to analyze the laminar, Newtonian, and unsteady flow into the microchannels. The computational domain was discretized using a tetrahedral mesh, where grids were refined near the walls to achieve accurate results. The governing Navier-Stokes and Convection-diffusion equations were discretized by the finite element method and solved using the commercial software COMSOL Multiphysics (Vers.5.2). In this work, the test runs were carried out with various grid sizes to ensure the grid independency of the results and the convergence criteria was set to $10^{-6}$.

## 3. Results and Discussion

The microfluidic-based placenta-on-a-chip was investigated numerically and experimentally. The numerical analysis was validated by comparing the simulation results to the diffusion rates measured from experiments. The rate of glucose transfer, the effects of flow rate, and membrane porosity (ε) on glucose diffusion across the membrane were studied. In this study, polycarbonate membrane with 400 nm pore size was used as a substitute for basement membrane. This pores size is significantly larger than in common basement membranes, for example glomerular basement membrane (pores ranging from 10 to 14 nm) [42]. As glucose molecules have much smaller size, the actual barrier for their transport are two layers of epithelial and endothelial cells on both sides of the membrane [35]. Thus, our approach for this particular case (small molecules transfer) disregards the size of the basement membrane pores.

### 3.1. Cell Culture in the Microfluidic Chip

**Figure 2:**
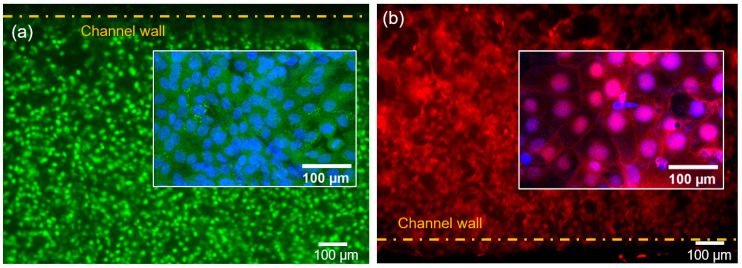
Cells in channels after 72 h of cell co-culturing. (**a**) Representative fluorescence image of human umbilical vein endothelial cells (HUVECs) stained with calcein-AM with the inset showing the nuclei stained with Hoechst 33342. (**b**) Representative fluorescence image of BeWo cells stained with CellTracker Orange Florescent Probe with the inset showing the nuclei stained with Hoechst 33342.

Cell confluency of 86 ± 3% and 90 ± 2% were achieved 72 h post cell seeding for HUVEC and BeWo cell layers, respectively. To verify the formation of intercellular junctions of the monolayers of BeWo and HUVECs cells, immunofluorescence assay was used. Figure 2a,b show representative images for calcein-stained HUVEC cells and CellTracker orange fluorescence-stained BeWo cells growing on each surface of the porous membrane. The nuclei of both cell types were stained with Hoechst 33342 (Fisher Scientific, Carlsbad, CA, USA, Cat. No. H3570). In the upper and lower channels, monolayers of BeWo and HUVECs formed a multi-layer membrane structure. Cell adhesion is vital for modeling the placental barrier and glucose transfer under flow condition. A high flow rate causes high shear stress on the walls, which could detach the cells from the membrane. An optimal flow rate in our experiment was found to be 50 μL/h, matching the physiological flow conditions in vivo [43]. This flow rate was used in the glucose transport experiments.

### 3.2. Glucose Transport across the Membrane

Molecular permeation in tissue is a dynamic process. Barrier permeability of the glucose across the barrier interface can be determined from Fick’s Law [44,45]

(7) $$P=\left(\Delta C\cdot V\right)/(C_{0}\cdot A\cdot t)$$

where P is solute permeability coefficient (cm/s), V is medium volume in the bottom channel, *t* is experiment time 2 h. ΔC is the solute concentration change from top channel to bottom channel, as measured through the two outlets. C_0_ is the initial concentration for top channel. The barrier permeabilities for the membrane with no cell layers, with cell monolayer cultured and co-cultured are compared. The glucose permeability through the bare membrane is higher than membrane with confluent monolayer cells. The permeability value for bare membrane was 4 times as high as the BeWo/HUVECs co-culture, being 1.65 × 10^−5^ ± 9.1 × 10^−7^ cm/s and 3.7 × 10^−6^ ± 7.3 × 10^−8^ cm/s, respectively.

**Figure 3:**
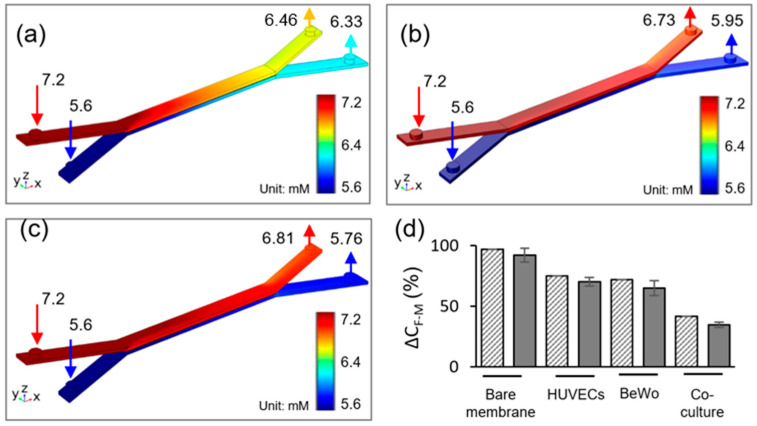
Glucose concentration profiles at flow rate of 50 μL/h in (**a**) bare membrane, (**b**) trophoblast monoculture, (**c**) trophoblast/HUVEC co-culture. (**d**) Comparison between experimental data and numerical simulation of glucose diffusion rate, ΔCF-M across the maternal-fetal interface for 2 h experiment time, under the 50 μL/h flow rate. Gray solid fill—experimental data, pattern fill—numerical simulation.

Glucose is a main source of energy to support fetus development during the pregnancy. Glucose transport in the placental barrier mimics was evaluated by the glucose concentration measurements from the upper and lower channel outflow. The simulated glucose concentration profiles after 2 h of flow under an equal inflow rate of Q1 = Q2 = 50 μL/h, are depicted in Figure 3. The inlet glucose concentrations were set to be C1 = 7.2 mM and C2 = 5.6 mM for top and bottom channels, respectively. These values were constant during the 2 h experiment, as verified by the glucose meter. Glucose diffusion across the bare membrane, HUVECs/BeWo cells monoculture, and co-culture are shown in Figure 3a–c, respectively. The absence of cells on the membrane results in enhanced glucose transfer across the membrane. It is obvious that cell monolayer connected in series with porous membrane contributed significant resistance to the molecular diffusion, causing a decrease in glucose transport across the barrier. Simulation results show that the glucose concentration in the outlets of the top and bottom channels are 6.73 mM and 5.95 mM, respectively (Figure 3b). The cell co-culture on the membrane further limited the molecular transport, where the glucose transfer is the lowest among the three conditions. In co-culture model, the glucose concentration in the outlets of top and bottom channels are 6.81 mM and 5.76 mM, respectively (Figure 3c).

Figure 3d demonstrates a comparison between the simulation and experimental results of the rate of glucose transfer across the different barrier models. In the bare membrane model, glucose transfer rate was measured as 93 ± 5.7%. When cells were cultured in the microdevice, the rate of glucose transfer decreased to 70 ± 6.1% and 63 ± 4.2% for HUVECs and BeWo cells, respectively. When BeWo/HUVECs co-culture was included in the model, the rate of glucose transport further reduced to be one third of the bare membrane model, being 35 ± 2.5%. The simulation results are in good agreement with the experimental results. The overall simulated glucose transfer rate is slightly higher than the experimental data. The small deviation may be attributed to the variations in the physical properties of cell culture medium, cell consumption, and channel roughness between the experiment and simulation.

### 3.3. Effects of Flow Rate on Glucose Concentration

**Figure 4:**
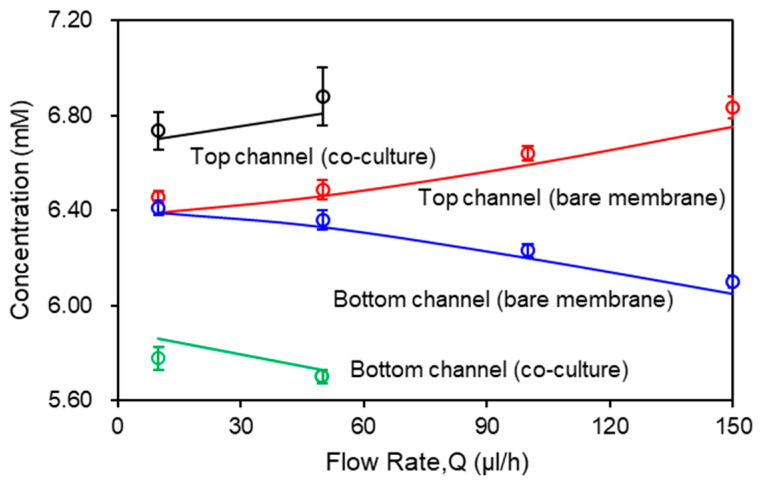
Glucose concentration as a function of flow rate. Computational results are represented by solid curves. Experimental data are represented by open circles in the corresponding color.

Flow rate in the channel is a dominant parameter that affects the molecular transport across the membrane. Figure 4 shows the effects of flow rate on the glucose transport in the bare membrane model and the co-culture model. The inlet glucose concentrations in the top and bottom channel were set as C1 = 7.2 mM and C2 = 5.6 mM, respectively. The flow rate varies from 10 µL/h to 150 µL/h. The glucose concentration was measured on both outlets of the micro channels (200 μm deep) with 0.01 membrane porosity and co-current flows condition. According to the conservation of mass law, the molecular losses in the top channel equals to the molecular gains in the bottom channel. At low flow rate (Q ≤ 50 μL/h), convection and diffusion mechanisms are comparable and decreasing the flow rate will also decrease the convective mechanism. At the lowest flow rate, Q = 10 μL/h, glucose concentrations are approximately equivalent due to complete mixing of the molecules between the two channels through the bare membrane. As flow rate increases, diffusion across the membrane decreases. This is reflected in the variation between the concentration profiles as measured in the outlets of the top and bottom channels, which increases with the flow rate. At the highest flow rate (Q = 150 μL/h), diffusion becomes almost negligible. In contrast, due to the resistance from the cell layers, the co-culture model shows a separation in the glucose concentration between the top and bottom channels at the lowest flow rate, Q = 10 μL/h, being 6.72 mM and 5.81 mM, respectively. When flow rate increases to Q = 50 μL/h, glucose concentration increases to 6.81 mM in the top channel and decreases to 5.76 mM in the bottom channel. The glucose concentration profiles in both models show close agreement between the numerical and experiemntal data.

### 3.4. Effects of the Membrane Porosity on Glucose Concentration

**Figure 5:**
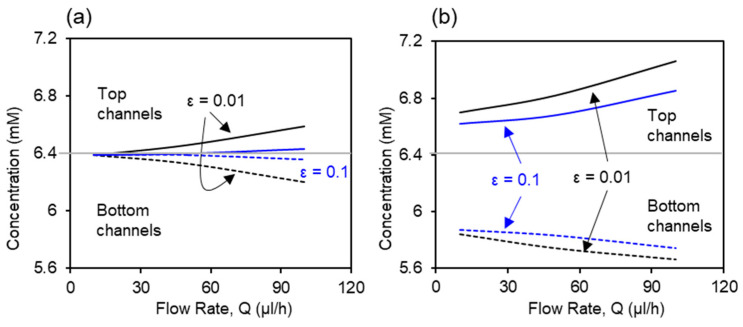
Effects of membrane porosity on the glucose transport in (**a**) bare membrane model and (**b**) co-culture model. Solid curves represent the glucose concentration in the top channels and dashed lines represent the glucose concentration in bottom channels.

Based on the above validated numerical model for placental barrier, the effect of membrane porosity on the glucose transport is investigated using numerical simulation. The inlet glucose concentrations in the top and bottom channel were set as the same as experiments, C1 = 7.2 mM and C2 = 5.6 mM, respectively. Glucose concentration values in the outlets of the top and bottom channels are extracted from simulation. Figure 5 compares the glucose concentration profiles in bare membrane and co-culture models with two porosity values, 0.01 and 0.1. Glucose concentration increases in the top channel and decreases in the bottom channel as the flow rate increases, disregarding the porosity value and cell presence. Figure 5a shows the profiles of the glucose concentration with two membrane porosity values for the bare membrane model. At the lowest flow rate (Q = 10 μL/h), complete mixing between the top and bottom channels were observed under both porosity values. The separation in the glucose concentration between the top and bottom channels is greater in the 0.01 porosity membrane than the 0.1 porosity membrane, due to the lower resistance to mass transfer from the higher membrane porosity. Figure 5b shows the profiles of glucose concentration in the co-culture model. Presence of the cells at the interface provided greater resistance to the glucose transport across the barrier comparing to the bare membrane condition. This acts together with the deviation in the cellular glucose consumption rates by the two cell types, leading to large separation between the glucose concentration in the top and bottom channels, even at the lowest flow rate of Q = 10 μL/h. The effects of membrane porosity are significant, where the higher membrane porosity, 0.1, facilitates mass transfer at all flow rates.

## 4. Materials and Methods

### 4.1. Cell Culture

Human trophoblast cell line (BeWo cells; ATCC^®^ CCL-98™) were cultured in Ham’s F-12K nutrient mixture (Corning Cat. No. 10-025-CV) with 10% fetal bovine serum (ATCC Cat. No. 30-2020) and 40 μg/mL gentamicin (GIBCO, Gaithersburg, MD, USA, Ref. No. 15-750-060). Human umbilical vein endothelial cells (HUVECs; ATCC^®^ CRL-1730™) were cultured in endothelial cell growth medium kit (EGM-2; Lonza, Alpharetta, GA, USA, Cat. No. CC-3162). The HUVEC and BeWo cells were cultured at 37 °C in 5% CO_2_ atmosphere to reach 90% confluence.

### 4.2. Microchip Design and Fabrication

The 3D placenta–on-a-chip device consists of a polycarbonate membrane and two Poly dimethylsiloxane (PDMS) microchannels (height: 200 μm; width: 1 mm) (Figure 1). The mold for casting the microfluidic channel was designed using SolidWorks (Autodesk, Inc., San Rafael, CA, USA) and printed using a clear resin with a 3D printer (Formlab Form 3). Standard soft lithography technique was used to cast the microfluidic channels from a PDMS mixture (weight ratio of 10:1 for base elastomer: curing agent). After degassing with vacuum pump, PDMS was poured onto the mold and allowed to cure in the oven at 65 °C for 3 h. Once the PDMS was fully cured, it was detached from the mold and cut into units of top and bottom channels. Biopsy punch of 1.0 mm diameter (Integra Miltex, Inc., Rietheim-Weilheim, Germany) was used to make the inlet and outlet holes for channels. The 3D micro device was assembled by bonding one top channel and one bottom channel to a polycarbonate membrane containing 400 nm pores (Global Life Sciences Solutions, Marlborough, MA, USA) using an adhesive PDMS/toluene mortar. To form a thin uniform layer of the adhesive mortar layer, 200 µL of PDMS and toluene mixture at a ratio of 5:3 in weight was poured on the glass coverslip and spin-coated at 1500 rpm for 60 s. A 4-h postbake at 60 °C was used to ensure firm bonding of the device. To facilitate injection of cell suspension into the channel, an adapter (Cole Parmer, Vernon Hills, IL, USA, Cat. No. UB-45518-00) was inserted to each inlet and outlet. All devices were sterilized with UV irradiation for 2 h.

### 4.3. Microfluidic Cell Culture

Human placental barrier consists of the trophoblastic epithelium, the chorionic connective tissue, and the fetal capillary endothelium. To simulate the placental barrier, BeWo cells and HUVECs were cultured on the top and bottom surfaces of the porous polycarbonate membrane, representing the epithelial layer and the endothelial layer, respectively. Before seeding the cells for culture, Type I collagen (GIBCO, Gaithersburg, MD, USA, Cat. No. A 1048301) was coated on the polycarbonate membrane to enhance cell attachment, following the manufacturer’s protocol. Then, a suspension of HUVECs (2 × 10^6^ cells per mL) were introduced into the bottom channel. The chip was immediately inverted to allow cells settle on the bottom surface of the membrane. The chip was incubated in CO_2_ incubator for 2 h, allowing the HUVECs attach to the membrane. After incubation, the chip was flipped and BeWo cells (6 × 10^6^ cells per mL) were introduced into the top channel for culture in CO_2_ incubator. Cell culture media for each cell type was changed daily in both channels.

Viability test of BeWo and HUVECs was performed using the Cell Tracker Orange fluorescent probes (Thermo Fisher Scientific, Waltham, MA, USA, Cat. No. C2927) and LIVE/DEAD™ Cell Imaging Kit (488/570), (Fisher Scientific, Carlsbad, CA, USA, Cat. No. R37601) at 72 h of incubation, according to the manufacturer’s protocol. Cell nuclei were also stained with Hoechst 33342 trihydrochloride (Fisher Scientific, Carlsbad, CA, USA, Cat. No. H3570) and observed under an Olympus IX81 fluorescent microscope.

### 4.4. Placental Barrier Permeability and Diffusion Rate

Glucose transfer across the placental barrier was measured three days post cell culture in the 3D microfluidic device. An initial glucose gradient, 30 mg/dL was created across the barrier. This was achieved by adjusting the glucose concentrations (both in physiological range) in the F12K medium to 130 mg/dL (7.2 mM) and in the EGM2 culture medium to 100 mg/dL (5.6 mM), supplied to BeWo layer and HUVECs layer, respectively. Medium flow was maintained at a rate of 50 µL/h for 2 h using a peristaltic pump (Harvard Peristaltic pump, P70), see Figure 1a. In each circulation, two tubings with inside diameter of 0.06 inch and length of 16 inch were used to connect the microfluidic channel and corresponding culture medium with the peristaltic pump. Then the outflow collected from lower and upper micro channels were sampled for glucose measurement via a GM 100 glucose meter (BioReactor Sciences, Lawrenceville, Georgia, USA). Glucose diffusion rate, ΔC_F-M_ across the placental barrier was calculated with the following equation,

(8) $$\Delta C_{F-M}=\Delta C_{F}/\Delta C_{M}$$

where ΔC_F_ and ΔC_M_ represent the change in the glucose concentration in bottom and top channel respectively, between each inlet and outlet. The results of glucose diffusion rate of the co-culture model of placental barrier were compared to results obtained in the other two conditions, including a bare membrane model and BeWo or HUVECs monoculture model.

## 5. Conclusions

In this paper, a microfluidics-based placenta-on-a-chip was developed to analyze the glucose transfer across the membrane. Barrier permeability and rate of glucose transfer were studied in this device. The results show that the glucose diffusion rate in co-culture cells model is smaller compared to monoculture and microdevice with no cells. To compare experimental and numerical results, a 3D simulation was carried out using commercial software (COMSOL 5.2) to analyze the unsteady flow into micro channels. Finally, the diffusion rates on experimental and simulation were compared together. The results show good agreement between experimental and numerical study. Effects of other parameters such as flow rate and membrane porosity on glucose transport across the placental barrier model were investigated by numerical simulations. The results show that the rate of glucose diffusion increases with membrane porosity and decreases with the flow rate.

Our microfluidics-based placenta-on-a-chip has potential to serve as a low-cost platform with a broad range of applications. For example, it can provide new opportunities to investigate placental transfer of various substances under different conditions. This microdevice can be used as an in vitro model to study the nutrient transfer across the maternal–fetal interface in placental malaria and contribute to better understanding and treatment of this disease.
